# Academy of Stomatology

**Published:** 1904-09

**Authors:** 

**Affiliations:** Academy of Stomatology


### ACADEMY OF STOMATOLOGY.

The regular meeting of the Academy of Stomatology of Philadelphia was held at its rooms, 1731 Chestnut Street, on the evening of January 26, 1904, Dr. L. Foster Jack, President, in the chair.

A paper, entitled " A Method for reproducing the Natural Contour of Artificial Teeth on the Lingual and Palatal Surfaces of Artificial Dentures," was read by Dr. William M. Fine.

(For Dr. Fine's paper, see page 569.)

A paper, entitled, " Lack of Dentition, with Presentation of an Illustrated Case, and the Exhibition of a Patient with Ankylosis of the Mandible," was read by Dr. William J. Roe.

### DISCUSSION OF DR. FINE'S PAPER.

*Dr. J. H. Gaskill.*—The method described by the essayist is certainly interesting, and his results are most beautiful from an artistic stand-point. The question arises whether the plate is not greatly weakened by cutting away so much of the palatal portion. According to the diagrams it is brought down very thin and at a short angle. I would like to ask whether there is not danger of breaking.

*Dr. Fine.*—The plate looks much thinner in the diagram than it is in reality. The joints between the teeth are much stronger than one would think. I have several patients wearing these, in both gold and vulcanite, and they say they would not care to change. The plate is very strong, as you can see by the samples passed around.

*Dr. G. Milliken.*—The process described is, from an artistic stand-point, very interesting, and I should think the results would be very beautiful. We cannot take too much pains in the making of fixtures for the mouth, and the nearer we approach to nature the more beautiful will be the result and the more agreeable to those who have to wear these appliances.

*Dr. R. H. Nones.*—While listening to the paper it seemed to me that the method was an old one, presented in a new form. I do not mean to detract, but would say that this method, with modifications, has been pursued for a number of years. The work is beautiful and artistic, and it is useful, and strength is not sacrificed to artistic effect when the work is properly done. The apparently weak plate shown in Diagram No. 4 is really much stronger than the one shown in Diagram No. 3. It is surprising how readily some of these thick plates break, because the strength is not put in the proper place.

Some will say they have not the time to do this work. If a man cannot take the time to do prosthetic dentistry, he had better let it be done by the men who can.

Instead of cutting the form of the vacuum chamber in the plaster impression, I prefer to inset the metal form upon the model, as I can judge better of its proper location.

Instead of covering the model with tin-foil, I make, when it is possible, a tin model, preferably a tin shell, which has the advantage of being readily removed from the plate, and is easily fastened to the articulator.

A peculiarity which I cannot explain is that rubber, when vulcanized upon tin, is totally different from rubber vulcanized in contact with plaster. It seems to lose its weight almost one-half.

I think the essayist is to be commended from the stand-point of doing well that which is worth doing at all. Prosthetic dentistry can be made quite as pleasurable as operative dentistry, provided it is well done and an interest taken in it.

*Dr. Fine* (closing).—I cover all of my models with tin-foil, thick on the outside and thin on the inside, and when possible I use a tin die. I use tin-foil because the latter is not always possible.

DISCUSSION OF DR. ROE'S PAPER.

*Dr. Roe.*—The anchylosis in this case is almost complete. The condition was recognized when the patient was quite young. I expect to operate in a few days and liberate the mandible.

*Dr. Darby.*—Did the boy ever have scarlet fever?

*Dr. Roe.*—No. He has had measles. He eats everything and talks well. The upper and lower gums are in contact in both buccal regions, but are not united, as I have passed a thin pliant instrument between them.

*Dr. Arnold.*—I cannot tell why there are no teeth in the case exhibited by Dr. Roe. This is a case of arrested development, and, like most of these cases, there is no known reason. I am sure there is nothing in the family history to account for it. Two more healthy, robust-looking people than the parents are not easily found.

*Question.*—Has a radiograph been taken?

*Answer.*—No; Dr. Roe has that in contemplation.

*Question.*—I would like to ask Dr. Roe whether anything is known in this case concerning prenatal maternal impressions?

*Dr. Roe.*—I made special inquiry, and the mother assured me that she had in every sense as normal gestation as with her other children. There was no abnormal condition during gestation, no fright or other strong prenatal impression.

*Dr. S. H. Guilford.*—I did not know that Dr. Roe was going to allude to a case which I observed some twenty years ago, but he has given some of the important facts regarding it. It is the only case I know of which was totally edentulous. The man never had any teeth, either deciduous or permanent. I know this, because I took the trouble to travel through the country where he lived and inquired of people who had known him from early childhood. I was unable to trace his ancestry or find out whether his peculiarities were transmitted. When he was in Philadelphia, twenty years ago, he was forty-eight years of age. I have brought with me the models taken from the impression made at that time. They look like those of the mouth of an old person. A strange thing was the ability of this man to eat. His meat was cut in rather small pieces, and these he would press between his tongue and the roof of his mouth and in this way he had developed a very muscular tongue. An aunt on the mother's side had a similar condition. Of the children whom I saw, the boys' dentures were normal. Of the daughters, one had fourteen teeth and one sixteen. They had four or five scattered teeth in the lower jaw, and the balance scattered about the upper jaw.

In collecting the cases for my paper read before the California Congress, I visited dime museums, boarding-houses, etc. Some cases reported as being edentulous turned out not to be.

Another point which interested me was that this boy has been troubled with ozæna. The patient that I had was also troubled with this condition.

In trying to make a scientific investigation of this kind we do not always come out exactly where we expect to. Ten years ago I thought that I was going to prove that where there is absence of hair there is absence of teeth. I found, however, that sometimes there is a deficiency of the one and abundance of the other, and the cases are so confusing that it is difficult to get satisfactory data.

*Dr. E. T. Darby.*—I have nothing special to say except to speak of models at the University of a case similar to that of this boy. There are, however, two permanent molars in the mouth. The boy had had no temporary teeth at all, but when about fifteen or sixteen years of age he erupted these two molars. From the models they look like second, instead of first, molars. The models were sent to me by a dentist in New York State. He said he had known the boy from the time he was an infant, and had watched the development of his mouth, and that these were the only teeth the boy had had.

*Dr. Guilford.*—Were they in the same jaw?

*Dr. Darby.*—No; in the opposite jaws. There was a fine alveolar ridge in the lower jaw. I corresponded with the dentist about the case, and he wanted to send him on, but the boy never came. Artificial teeth were made, but the lad got along so well without them that he did not care to be bothered. He could eat fairly well.

It is said that the absence of hair and absence of teeth are noted together. The man whom Dr. Guilford speaks of I believe never perspired, and he slept in the cellar. I have known one or two persons who practically never perspired, but they were not edentulous.

*Dr. G. Milliken.*—It seems to me that something might be found out about such cases. An exhaustive examination of the blood, saliva, a section of the skin, or, if possible, a portion of bone, possibly taken from the jaw, might throw some light on the question.

*Dr. Roe* (closing).—I am gratified with the interest the members of the Academy have taken in this boy's condition; and, following out Dr. Milliken's suggestion, I certainly think that a histological examination of the skin would be interesting. Whether an examination of the blood and saliva would reveal anything is a question.

Much waste product of metabolism is eliminated by the normal skin. If the skin be not capable of this normal function, there must be a very great deal of work thrown upon the other excretory organs, such as the lungs and kidneys.

(Replying to Dr. Milliken.) I have inquired into the family history for evidence of syphilitic infection, tuberculosis, or chronic alcoholism, and have found no evidence of any of the three conditions. The family shows every evidence of normal good health and generally good development.

OTTO E. INGLIS,
*Editor Academy of Stomatology.*

